# Psychosocial and clinical factors associated with depression among individuals with diabetes in Bahir Dar City Administrative, Northwest Ethiopia

**DOI:** 10.1186/s12991-020-00267-6

**Published:** 2020-03-11

**Authors:** Teshager Woldegiorgis Abate, Haileyesus Gedamu

**Affiliations:** Department of Adult Health Nursing, College of Medicine and Health Science, Bahir Dar University, Bahir Dar, Ethiopia

**Keywords:** Diabetes, Depression, Adult patient, Co-morbidity, Factors

## Abstract

**Background:**

In a context of the potential epidemic nature of both diabetes mellitus (DM) and depression had negative effects in cases of disability and mortality. Coexisting depression had been linked to morbidity and mortality in people with diabetes. Therefore, this study aimed to identify psychosocial and clinical factors associated to develop depression symptoms in diabetes patients.

**Methods:**

A systematic institution based cross-sectional study design was conducted from 1st March to 8th April 2016 among adult diabetes patent. Depression status was assessed by the Patient Health Questionnaire-9 (PHQ-9). Data were analyzed by logistic regression and at P < 0.05 with 95% CI was considered statistically significant.

**Result:**

A total of 416 patients were included in this studied. Based on PHQ-9, self- reported depression symptom was 29.3% [95% CI 25.2, 33.4]. In the performed statistical evaluation, patients whose age was between 45 and 54 years old (AOR = 3.88; 95% CI 1.36, 11.08); being female (AOR = 2.43; 95% CI 1.29, 4.58); who has poor social support (AOR = 6.08; 95% CI 2.98, 12.40); and who has high fear of kidney problems secondary to DM (AOR = 6.12; 95% CI 1.75, 21.23) were statistically associated with depressive symptoms in diabetes patients.

**Conclusion:**

This study demonstrated that complication fears, social support, being female and sexual dysfunction were found significantly associated with co-morbid depression in diabetes. Therefore, availed a social network of family and friends; providing diabetes education to address the patient’s fear of complications; and considered a sexual function is an integral part of overall health in diabetic patients.

## Background

Diabetes mellitus (DM) is a chronic metabolic disorder. It affects the productive age group. Globally, an estimated 422 million. adults were living with diabetes in 2014. By 2030 this will have risen to 552 million. people. DM was a cause of 4.6 million. deaths in 2011 [1, 2]. Studies showed that diabetes and depression occur together approximately twice as frequently as would be predicted by chance alone [3]. Depression is a common mental disorder characterized by sadness, loss of interest or pleasure, feelings of guilt or low self-worth, disturbed sleep or appetite, feelings of tiredness, and poor concentration [4]. Currently, at a global level, over 350 million. people are suffering from depression and its leading cause of disability [5].

It is believed that the relationship of diabetes to depression is bidirectional. That is why depression can lead to diabetes and conversely, diabetes could facilitate the emergence of depression [6]. Diabetes mellitus can cause psychological, emotional, social and psychosexual problems. Depression is also a risk factor as well as complications of diabetes [5, 6]. Individuals with diabetes have a 2- to 3-fold increased risk for depression. Depression affecting approximately one in every five diabetes patients compared to the general population [5, 7]. Co-morbid diabetes and depression are a major clinical challenge as a treatment outcome of both conditions are worsened by one the other [3].

Previous studies documented that socio-demographic, clinical and psychosocial factors contributing to the development of co-morbid depression among diabetes patients. Socio-demographic factors that were significantly associated with depression in people with diabetes were; older age, lower socioeconomic status, monthly household income and ethnicity [8–10], level of education [12], female gender [13] and being single [14]. A clinical factors which were linked to depression in diabetic patients were presence of complication [12], co-morbidities, duration of treatment and treatment with insulin [10], duration of illness more than 10 years [15], limb problems [16], presence of ≥ 3 diabetic complications, and negative life events and poor social support [17], total number of life events were statistically significant predictors of depression in diabetes patient [18].

Behavioral related factors that were significantly influenced co-morbid depression in diabetes patients were also: social inhibition and negatively affective life [19], poor social support [14, 20], physical quality of life [21], future diet behavior, physical activity and glucose testing [22]. A literature review reported that diabetes individuals who suffer from depression, DM related complications, and lack of social support were worse general health [23].

Psychosocial and clinical factors on co-morbid depression influence living with diabetes, both type 1 and type 2 [24]. Although the above factors were associated with depression, most of these studies were based on in developed countries, with only some exceptions. Therefore, these findings cannot be generalized to Ethiopia, especially in the study area. Thus, our study aimed to identify psychosocial and clinical factors associated with co-morbid depression among diabetes patients treated at the Felege Hiwot Referral Hospital (FHRH), Bahir Dar, Northwest Ethiopia.

## Methods

### Study setting and population

A facility-based cross-sectional study was conducted from 1st March to 8th April 2016 at FHRH. FHRH is found in Bahir Dar city administration, Northwest Ethiopia. The hospital was opened for 24 h for emergency service. It provides promotive, preventive, curative and rehabilitative services.

The service was rendered by the physician and nurses. Around 1484 patients were registered for follow-up in the previous year. Those with diabetic patients were used to collect their medication on every 2 to 3-month basis. In the outpatient chronic follow up department, approximately 300 adult diabetes mellitus patients were seen weekly. All diabetic patients aged 18 years and older; and who had been on regular follow up treatment for DM regardless of ethnicity and religion were participating in the study. Hearing and/or verbalize impairment, serious illness and newly diagnosed during data collection were excluded from the study.

Participants were hearing and/or verbalizing impairment; those who did not provide coherent answers to the demographic questions. The participant was defined as a serious illness to difficult participate who had a severe medical or psychiatric illness that made it impossible to complete the study questionnaire in one setting, who were requested to terminate the interview for medical reasons, or the data collectors were perceived that the participant was too sick to participate. Participants were newly diagnosed means that they diagnosed before 6 months. This was by considering these participants could be perceived them an inadequacy awareness of the disease to get enough information.

### Sample size determination and sampling procedure

The sample size was calculated by assuming a 5% marginal error (d), 95% CI (alpha = 0. 05) and 43.6 the proportion of depression among DM patients [25]**.** Based on this assumption, the sample size was calculated by the single population proportion formula: n = (Z1t − α/2)2 p (1 − p)/d2; this yields an initial sample size of 378. By considering a 10% non-response rate, 416 diabetic patients were planned and included in the study. A list of diabetic patients was obtained from the outpatient follow up department and used as a sampling frame.

The patients’ records were listed in the follow-up appointment order. Then, the respondents were selected by using a systematic random sampling technique. Based on the decision to collect data for 1 month, the sampling interval was determined by dividing the expected number of diabetic patients per month (1320) into the sample size (416) which gives a sampling interval of three. Thus, every third patient coming to a follow-up service was interviewed until the total sample size was reached.

Data were collected using a face to face interview questionnaire to acquire demographic information; fear of diabetes complications [by using revised fear of complications questionnaire (FCQ)]; and social support (by using Oslo 3-items Social Support Questionnaire). To assess recent clinical related data: fasting plasma glucose level, co-morbidity, diabetes-related complications, duration of diabetes, treatment modality and duration of treatment were collected from the patients’ records by using checklists. The sum of the Oslo 3-items social support scale was ranging from 3 to 14. When social support scale scores were ranged from 3 to 8 is categorized as “poor support”, 9–11 is “moderate support” and 12–14 is “strong support” [26, 27].

Fear of DM-rerated complication questionnaire was assessed specific fears (e.g. blindness, kidney problems, and heart disease, etc.), lifestyle fears, fears of hypoglycemia and future quality of life. It was a self-assessment questionnaire designed to measure fear of complications in diabetes. The items were rated on a four-point scale (0–3), where 0 denotes a low fear of complications and 3 refers to high fear [28]. Perceived low fear of complication was recorded “no” fear of complication whereas perceived high fear of complication also recorded as “yes” had a fear of complication for each item.

Patients with established diabetes mellitus were evaluated for depression by interviewing nine-item of Patient Health Questionnaire nine (PHQ-9) adapted from Pfizer Inc. using local language (Amharic). PHQ-9 is a criteria-based diagnostics tool of depressive disorders and it was also a reliable and valid measure of depression severity. It is a brief questionnaire that scores each of 9 DSM-IV criteria for depression as “0” (not at all) to “3” (nearly every day). PHQ-9 score ≥ 10 had a sensitivity of 88% and specificity of 88% for major depression [29]. PHQ-9 scores of 5, 10, 15, and 20 represent mild, moderate, moderately severe and severe depression, respectively [29, 30].

Since the questionnaire was prepared in English, it was translated into the Amharic language for appropriate and easiness in interviewing the study subjects in the Amharic language. The psychometric properties and cross-cultural validation in the local language of PHQ-9 were validated in Ethiopia [31]. To assure data quality, two weeks before the actual data collection, the questionnaire was pre-tested on 5% of the sample among diabetes patients who were not included in the main study area. Data were collected by four trained, diploma nurses and two supervisors (BSc nurse). Additionally, on each data collection day, the collected data were reviewed and checked for its completeness by principal investigator and supervisors.

### Data analysis

Data were entered and edited into EPI-data Version 3.1. The entered data was analyzed by using Statistical Package for Social Science (SPSS) for Windows program version 20. The association was investigated to assess the association between dependent and explanatory variables using binary logistic regression. All explanatory variables with p-value ≤ 0. 20 in the bivariate logistic analysis were fitted into a multivariate logistic regression to identify independently associated factors in the final model. The degree of association was interpreted by using Odds Ratio with 95% confidence interval. The p-value < 0.05 was considered statistically significant.

## Results

### Socio-demographic characteristics

Four hundred and sixteen study participants were interviewed with a response rate of 100%. Of those, 290 (69.7%) were married, and 120 (28.8%) were governmental employees. Regarding age distributions, the mean and Standard Deviation (SD) age of the participants was 45.5 ± 16.7 years old. Likewise, the median family monthly income of the study participant was 2000 Ethiopian Birr (ETB) (≥ 1401) (Table 1).

**Table 1:** Socio-demographic characteristics of study participants at Felege Hiwot referral hospital, Bahir Dar, Northwest Ethiopia, 2016 (n = 416)

Variable	Category	Frequency	Percent
Sex	Male	242	58.2
	Female	174	41.8
Current age	18–34	124	29.8
	35–44	81	19.5
	45–54	82	19.7
	≥ 55	129	31.0
Residency	Urban	316	76.0
	Rural	100	24.0
Marital status	Single	65	15.6
	Married	290	69.7
	Divorced	32	7.7
	Widowed	29	7.0
Educational status	Can’t read and write	124	29.8
	Read and write	47	11.3
	Primary school (1–8)	52	12.2
	Secondary school (9–12)	84	20.2
	College/University	109	26.2
Occupational status	House wife	48	11.5
	Farmer	103	24.8
	Merchant	65	15.6
	Governmental employee	135	32.5
	Private company employee	65	15.6
Average monthly income	≤ 650	111	26.7
	651–1400	101	24.3
	≥ 1401	204	49.0

### Clinical characteristics of study participant

About 238 (57.2%) patients were found to have type-II DM. Patients with type-I DM were taken the injectable form of treatment (insulin). Out of 416 participants, 70 (16.8%) participants had a cardiovascular disease (like hypertension) which was evidenced by a review of patients’ cards. Three hundred nineteen (76.7%) and 321 (77.2%) were living with diabetes mellitus and taking physician-prescribed medication at least for 8 years respectively (Table 2).

**Table 2:** Clinical characteristics of study participants at Felege Hiwot referral hospital, Bahir Dar, Northwest Ethiopia, 2006 (n = 416)

Variable	Category	Frequency	Percent
Types of diabetes mellitus	Type 1	178	42.8
	Type 2	238	57.2
Diabetes treatment regime	Oral hypoglycemic and insulin	60	14.4
	Oral hypoglycemic agent only	124	29.7
	Single insulin only	232	55.8
Duration of DM (years)	≤ 8	319	76.7
	9–16	80	19.2
	≥ 17	17	4.1
Duration of DM Rx (years)	≤ 8	321	77.2
	9–16	75	18.0
	≥ 17	20	4.8
Comorbid disease	Cardiovascular disease	70	16.8
	Respiratory disease	9	2.2
	Neurological disease	12	2.9
	Renal disease	11	2.6
	Others disease	13	3.1
Complication of diabetes	Diabetic retinopathy	53	12.7
	Diabetic nephropathy	8	1.9
	Diabetic neuropathy	14	3.4
	Sexual dysfunction	19	4.6
Three consecutive average measurement of fasting plasma glucose level	> 126 mg/dl	262	63.0
	< 72 mg/dl	13	3.1
	72 mg/dl–126 mg/ dl	141	33.9

*Rx* treatment, *DM* diabetes mellitus, *mg/dl* milligram per deciliter

#### Psychosocial factors attribute to depression in the study participants

Of the total study participant, 213 (51.2%) were reported that they had poor social support. Regarding perceived fear of complication, 222 (53.4%) of respondents had fear of long-term diabetes complication, 109 (26.2%) participants were reported worrying about future problems when their blood sugars were erratic and 171 (41.1%) were worried about losing their eyesight because of diabetes related-complication (Table 3).

**Table 3:** Psychosocial characteristics of study participants at FHRH, Bahir Dar, Northwest Ethiopia, 2016 (n = 416)

Variables	Categories	Frequency	Percent
Social support^a^	Strong support	71	17.1
	Moderate support	132	31.7
	Poor support	213	51.2
Burden of medication	≤ 3	346	83.2
	≥ 4	70	16.8
I feel afraid of long-term complications of diabetes^a^	High fear	222	53.4
	Low fear	194	46.6
I am scared that diabetes could affect my feet	High fear	193	46.4
	Low fear	223	53.6
I am afraid I may need kidney dialysis one day	High fear	139	33.4
	Low fear	277	66.6
I worry about losing my eyesight because of diabetes	High fear	171	41.1
	Low fear	245	58.9
I worry diabetes increases chances of heart disease	High fear	122	29.3
	Low fear	294	70.7
Afraid of developing long-term complications as a result of frequent high blood sugars	High fear	128	30.8
	Low fear	288	69.2
I am afraid I will develop kidney problems one day	High fear	110	26.4
	Low fear	306	73.6
I worry that I might be at higher risk for having a stroke	High fear	94	22.6
	Low fear	322	77.4
Worry about your future health?	High fear	108	26.0
	Low fear	308	74.0
Worry about future when your blood sugars are erratic	High fear	109	26.2
	Low fear	307	73.8

^a^Social support was assessed by using Oslo 3-items Social Support Questionnaire and fear of complication was measured by revised fear of complications questionnaire (FCQ)

#### Factors associated with depression

The multivariate logistic regression analysis showed that participants with age 45–54 (AOR = 3.88; 95% CI 1.36, 11.08), being female (AOR = 2.43; (95% CI 1.29, 4.58); who had sexual dysfunction (AOR = 9. 52; 95% CI 2.19, 41.35); those who had moderately support in social life (AOR = 6. 49; 95% CI 2.83, 14.87); who had socially poor supported (AOR = 6. 08; 95% CI 2.98, 12.40); those who were worried about heart disease due to diabetes (AOR = 6. 49; 95% CI 2.22, 18.93) and who had a high fear of kidney problem in their future life (AOR = 6. 12; 95% CI 1.75, 21.23) were more likely had co-morbid depression disorder (Table 4).

**Table 4:** Logistic regression on the impact of selected socio-demographic, clinical and psychosocial factors, and depression symptoms among diabetes at FHRH, Bahir Dar, Northwest Ethiopia, 2016 (n = 416)

Variable categories	Depression (n)				COR (95% CI)		AOR (95% CI)
Age	Yes		No				
18–34	37		87		1.53 (0.87, 2.71)		1.83 (0.69, 4.88)
35–44	24		57		1.52 (0.81, 2.86)		4.22 (1.65, 10.76)
45–54	33		*49*		*2.43 (1.32, 4.46)*		*3.88 (1.36, 11.08)*
≥ 55*	28		101		1.00		1.00
Sex							
Male*	61		181		1.00		1.00
Female	61		*113*		*1.60 (1.05, 2.45)*		*2.43 (1.29, 4.58)*
Sexual dysfunction							
Yes	*11*		*8*		*3.54 (1.39, 9.04)*		*9.52 (2.19, 41.35)*
No*	111		286		1.00		1.00
Social support							
Strong support*	25		188		1.00		1.00
Moderate support	*55*		*77*		*10.89 (5.78, 20.47)*		*6.49 (2.83, 14.87)*
Poor support	*42*		*29*		*5.37 (3.12, 9.24)*		*6.08 (2.98, 12.40)*
I worry that having diabetes increases the chances of heart disease							
High fear	*74*		*48*		*7.90 (4.90, 12.73)*		*6.49 (2.22, 18.93)*
Low fear*	48		246		1.00		1.00
Afraid to develop kidney problems one day							
High fear	*69*		*41*		*8.03 (4.94, 13.07)*		*6.12 (1.75, 21.23)*
Low fear*	53		253		*1.00*		*1.00*

* Reference category; p-value = significant, < 0.05; *COR* Crude Odds Ratio, *AOR* Adjusted Odds Ratio

Patients who were 45–54 years had a significantly higher risk of having depression symptoms than those ages less than 45 and greater than 55 years (AOR = 3.88; 95% CI 1.36, 11.08). Females were 2.43 times more likely to develop depressive symptoms as compared to males (AOR = 2.43; (95% CI 1.29, 4.58). Participants who were suffering from sexual dysfunction 9.52 times more likely attack by depression symptoms as compared to those who had no sexual problems (AOR = 9. 52; 95% CI 2. 19, 41.35). There were also significant difference regarding social support; moderate, socially supported were 6.49 times more likely to develop depressive symptoms as compared to those strongly supported (AOR = 6. 49; 95% CI 2.83, 14.87). Patients who had a high fear for kidney problems related to diabetes were 6.12 times more likely to develop depressive symptoms as compared to those who had low fear (AOR = 6.12; 95% CI 1.75, 21.23) (Table 4).

## Discussion

To our knowledge, there is high impact of depression and co-morbid diabetes and its importance as a public health problem, little is known about the existence of depression in people with diabetes in the Amhara region, especially in Bahir Dar city. Therefore, this study aimed to assess psychosocial and clinical factors associated with depression among diabetes patients attending FHRH, Bahir Dar Northwest Ethiopia. In this study, age was the significantly associated depression symptom in diabetes patients. The risk factors for depression as per the bivariate analysis in this study were age, being female, presence of sexual dysfunction, moderate and poor social support, high fear of developing cardiac and kidney diseases for their future life.

In this study, age (45–54 years old) 3.88 times has a risk of developing depressive symptoms than those whose age was greater than 54 and less than 45 years old. This finding was alongside with other previous works done in different countries [9–11]. In this study, depression was significantly associated with the female gender that was 2.43 times has a risk to develop an attack of depressive symptoms than males. This finding confirms other studies done in Karnataka, Southern India and Peshawar [10, 32, 33].

The results of this study indicated that moderate and poor social support were significantly associated with depression symptoms. Participants who had a lack of social support from their families, neighbors, and friends were around 6.4 times more vulnerable to develop depression symptoms than participants who had strong social support. This finding was in agreement with studies conducted in Iran [14, 20], Croatia [21], Korea [34] and Addis Ababa [17]. This might be due to the effect of social support on emotional well-being (as in receiving love and empathy) and practical help (like the gifts of money, assistance with care). Hence, social support can come from social connectedness, feeling needed, a reassurance of self-worth, reliable support, advice and information, and physical and material assistance.

In our study, depression symptoms 9.52 times increased with the presence of sexual dysfunction in diabetes patients. This finding was supported by other previous studies that reported the presence of complications was strongly associated with depression symptoms in diabetes patients [10], the burden of a lifelong disorder of sexual problems [6]. The nature of diabetes mellitus is producing hyperglycemia, as well as angiopathy, vasculopathy, and neuropathic complication leads to sexual dysfunction in both genders [35]. This might be due to the pathophysiological effect of DM on sexual function through changes in blood supply to the urogenital tissue, reduce to genital lubrication, inability to achieve orgasm, dyspareunia and reduction in libido [36].

But other diabetes clinical risk factors that were significantly associated in varying degree with depression in people with diabetes includes hypertension and microvascular complication [37], diabetic nephropathy [17], physical activities, glucose testing [22], presence of co-morbidities, longer duration of treatment, treatment with insulin [10] and elevated fasting blood sugar level, physical disability and lack of a physician’s advice about lifestyle modifications [32]. Those variables in our study did not significant risk factors to provoke the attack of depressive symptoms in DM patients. This discrepancy might be due to variations in study design and setting, self-care characteristics of respondents, a therapeutic relationship of care provider, sedentary lifestyle of the participants and selection method of respondents.

In this study, high fear of future complication was found to be independently associated with developing depression in diabetes participants. Perceived high fear of cardiac complication with the adjusted odds ratio of 6.49 (2.22, 18.93), and perceived high fear of kidney complications with the adjusted odds ratio of 6.12 (1.75, 21.23) was strongly associated with developing depression. However, other studies were done in Karnataka [10], Iran [9], India [32] and Addis Ababa [17] which found no significant association in terms of fear of complications in diabetes patients. This discrepancy was might be due to almost all other studies that did not investigate the fear of complications. Another possible reason might be earlier studies focused on only demographic, clinical and psychosocial factors related to co-morbid depression in diabetes.

## Conclusion

In the current study, clinical factors (sexual dysfunction, sex, and age) and psychosocial factors (social support, fear of complication) were important factors significantly associated with co-morbid depression with patients living diabetes, both type 1 and type 2. To be promoted to optimal medical outcomes and psychological well-being, patient-centered care is essential. Therefore, the health care providers should be availed of a social network of family and friends (because family and friends are a significant source of support for coping mechanisms); providing diabetes education to address patient’s fear of long-term complications by fear reduction and promote survive skill. Sexual function also should be considered an integral part of overall health in diabetic patients.

### Strength

The study could be considered as a base for further similar and large-scale studies. The strengths of this study also a significant contribution to the body of knowledge in general and more specifically for health professionals and the patients themselves. Additionally, rather than having to rely on self-report, we were able to use information from patients’ medical diaries to gather information about the presence of diabetes complications, co-morbidity, and FBS levels. Also, we were using a reasonable sample size.

## Limitation

Due to the cross-sectional design of this study, it is not possible to draw any conclusion regarding causality. We also assessed level depression and other independent variables by using self-reported question turns to limit this study. Carefully analyzing longitudinal data and mixed design both qualitative and quantitative approaches are the best alternative in the future.

## Data Availability

Detailed data will not be shared due to the confidential nature of the data.

## Abbreviations

AOR: Adjusted odd ratio
CI: Confident interval
DM: Diabetes mellitus
DSM-IV: Diagnostic and Statistical of Mental Disorder-5
FCQ: Fear of Complication Questionnaires
FHRH: Felege Hiwot Referral Hospital
PHQ-9: Patent Health Questionnaires Nine
